# *Eugenia jambolana* (Java Plum) Fruit Extract Exhibits Anti-Cancer Activity against Early Stage Human HCT-116 Colon Cancer Cells and Colon Cancer Stem Cells

**DOI:** 10.3390/cancers8030029

**Published:** 2016-02-26

**Authors:** Venkata Charepalli, Lavanya Reddivari, Ramakrishna Vadde, Suresh Walia, Sridhar Radhakrishnan, Jairam K. P Vanamala

**Affiliations:** 1 Departmetn of Food Science, The Pennsylvania State University, University Park, PA 16802, USA; vxc166@psu.edu (V.C.); vrkrishna70@gmail.com (R.V.); sxr54@psu.edu (S.R.); 2 Departmetn of Plant Science, The Pennsylvania State University, University Park, PA 16802, USA; lur15@psu.edu; 3 Division of Agricultural Chemicals, Indian Agricultural Research Institute, New Delhi 110001, India; suresh_walia@yahoo.com; 4 The Pennsylvania State Hershey Cancer Institute, Penn State Milton S Hershey Medical Center, Hershey, PA 16802, USA

**Keywords:** colon cancer stem cells, java plum, anthocyanins, proliferation, apoptosis, stemness

## Abstract

The World Health Organization predicts over a 70% increase in cancer incidents in developing nations over the next decade. Although these nations have limited access to novel therapeutics, they do have access to foods that contain chemopreventive bioactive compounds such as anthocyanins, and as such, consumption of these foods can be encouraged to combat cancer. We and others have previously characterized the anti-colon cancer properties of dietary anthocyanins from different sources. *Eugenia jambolana* (Java plum) is a tropical medicinal fruit rich in anthocyanins, however, its anti-colon cancer properties are not well characterized. Furthermore, recent evidence suggests that colon cancer stem cells (colon CSCs) promote resistance to chemotherapy, relapse of tumors and contribute to poor prognosis. The objectives of this study were to 1) characterize the anthocyanin profile of Java plum using HPLC-MS; and 2) determine the anti-proliferative (cell counting and MTT) and pro-apoptotic (TUNEL and caspase 3/7 glo assay) properties of Java plum fruit extract (JPE) using HCT-116 colon cancer cell line and colon CSCs (positive for CD 44, CD 133 and ALDH1b1 markers). HPLC-MS analysis showed that JPE contains a variety of anthocyanins including glucosides of delphinidin, cyanidin, petunidin, peonidin and malvidin. JPE anthocyanins suppressed (*p* < 0.05) proliferation in HCT-116 cells and elevated (*p* < 0.05) apoptosis in both HCT-116 cells and colon CSCs. JPE also suppressed the stemness in colon CSCs as evaluated using colony formation assay. These results warrant further assessment of the anti-cancer activity of JPE, and its molecular mechanisms using pre-clinical models of colon cancer.

## 1. Introduction

Colon cancer is the second leading cause of cancer related deaths in the United States. For the year 2014, the American Cancer Society estimated that there would be about 136,803 new cases and 50,310 deaths due to colon cancer [1]. Colon cancer is caused by a step-wise accumulation of mutations in tumor suppressor and oncogenic genes, resulting in the formation of polyps which ultimately leads to adenocarcinoma [2]. There is increasing evidence that most cancers including colon cancer have a hierarchy of cells with cancer stem cells (CSCs) forming the core and sustaining the growth of the tumor [3]. CSCs including colon CSCs mimic the functionality of normal adult stem cells maintaining their un-differentiated state while dividing non-symmetrically [4]. They are also resistant to conventional therapies, thus leading to relapse of cancer in most patients [5,6]. Agents that target CSCs could be more efficacious and aid in preventing relapse.

Geographic differences in colon cancer rates and temporal changes in risk among immigrant populations suggest that diet and lifestyle strongly influence the occurrence of colon cancer. Granting research is still accumulating on the role of specific dietary elements on colorectal cancer risk, current research indicates that higher intake of certain diets including high in fat or red meat and lower intake of diet rich in fruits and vegetables is linked to a higher risk for colon cancer. However, unlike most cancers, colon cancer has a long latency period before it is detected (such as aberrant crypts) [7]. There is increasing evidence of the preventive/protective role of dietary bioactive compounds such as anthocyanins from fruits, vegetables, and herbs against a variety of cancers including colon cancer [8,9]. Individual anthocyanins, food-derived anthocyanin extracts and consumption of anthocyanin-rich foods exhibit anti-cancer properties in both *in vitro* and *in vivo* studies [10,11,12]. We have previously shown that anthocyanins from potato extracts suppressed cell proliferation and induced apoptosis in early (HCT-116) and advanced (HT-29) human colon cancer cell lines [13]. However, there is a dearth of data on the anti-cancer properties of anthocyanins against CSCs including colon CSCs.

The World Health Organization (WHO) has predicted that there will be 70% increase in cancer incidence in the developing countries [14]. More than 60 percent of the world’s new cancer cases occur in Africa, Asia, and Central and South America; 70 percent of the world’s cancer deaths also occur in these regions [15]. Although these nations have limited access to latest pharmaceutical drugs, people in these countries have access to foods that contain chemopreventive bioactive compounds such as anthocyanins, and as such, consumption of these foods can be encouraged to combat/prevent cancer. In the US and other developed countries, there is an increased public awareness of complementary and alternative medicinal approaches for chronic disease prevention, including different cancers. Although research on targeted pharmacological approaches is growing, the risk for different cancers does not seem to subside. National expenditures for cancer care in the United States totaled nearly $125 billion in 2010 and could reach $156 billion in 2020 [16]. Hence, dietary approaches for cancer prevention, including identification of new or development of existing dietary bioactive compound-rich foods are required.

The native Indian tree, *Eugenia jambolana* (common name: Java Plum) is found widely in the Asian sub-continent and other tropical regions of the world [17]. In the United States, *Eugenia jambolana* is found in Florida and Hawaii (USDA Natural Resource Conservation Service Plant Database). This underutilized tropical evergreen tree yields small purple ovoid fleshy fruits with an astringent taste. This fruit is valued for its diverse chemical constituents as well as medicinal and therapeutic properties [18,19]. In traditional Indian medicine, both the fruit pulp and seed extracts have a long history of medicinal use and they have been extensively studied for their anti-diabetic properties [20,21]. Previous studies have identified the major anthocyanins in Java plum fruit pulp/skin as diglucosides of delphinidin, petunidin and malvidin [17,22,23]. These anthocyanins are responsible for imparting the ripened fruit its bright purple color. Java plum fruit extract (JPE) has been shown to exhibit anti-proliferative and pro-apoptotic effects in estrogen dependent/aromatase positive, and estrogen independent breast cancer cells [23,24]. However, there is a lack of literature on the anti-colon cancer properties of anthocyanin-rich Java plum, particularly given the strong evidence of anti-cancer effects of dietary anthocyanins [11]. Furthermore, evidence of the effect of anthocyanin-rich foods against colon CSCs remains elusive. Thus, the current study was conducted to 1) characterize the anthocyanin profile of JPE, and 2) determine the anti-cancer properties of the JPE on HCT-116 and colon CSCs.

## 2. Results and Discussion

### 2.1. Evaluation of the Bioactive Compound Profile in JPE

**Figure 1:**
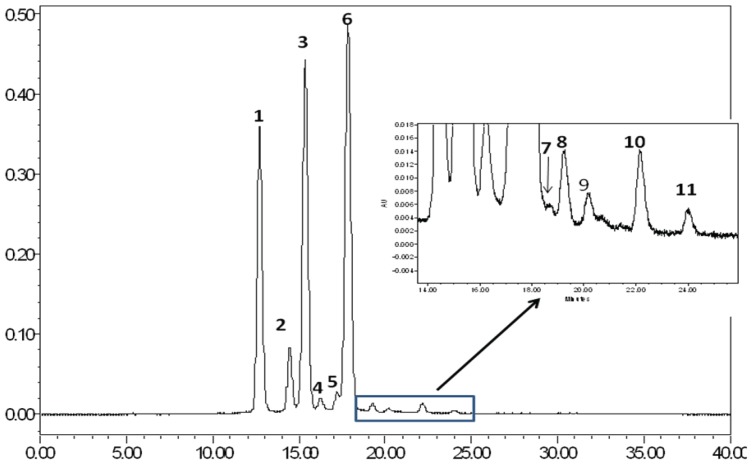
High Performance Liquid Chromatography (HPLC) chromatogram of Java plum fruit extracts (JPE) anthocyanins.

Liya Li, *et al.* [25] previously have reported the anthocyanin profile of JPS. They also reported that concentration and types of anthocyanins in JPE differs from the geographical region of the berries. Five types of anthocyanins––delphinidin-diglucoside, cyanidin-diglucoside, petunidin-diglucoside, peonidin-diglucoside, and malvidin-diglucoside were identified in their paper using HPLC and LC-MS. In our study, we also found the five reported anthocyanins using HPLC-MS (Figure 1 and Table 1). On the basis of mass spectral data, the three major anthocyanin peaks were identified as delphinidin-3, 5-diglucoside (1), cyanidin-3, 5-diglucoside (2), and petunidin-3, 5-diglucoside [26]. The remaining six minor anthocyanin constituents were similarly characterized as diglucosides of peonidin (4), and malvidin (5), and monoglucosides of delphinidin (6), cyanidin (7), petunidin (8) and malvidin (9). This may suggest that composition of JPE may be similar across locations, however the content might be different. In addition to anthocyanins, other class of phenolic compounds identified in JPE includes flavonols (Quercetin, Myricetin, Kaempferol, Luteolin, Isorhamnetin), flavanones (Naringenin) and stilbenoid (Resveratrol). All these compounds have been shown to exhibit anti-cancer properties [27,28].

### 2.2. JPE Suppressed Proliferation in HCT-116 Cells

**Figure 2:**
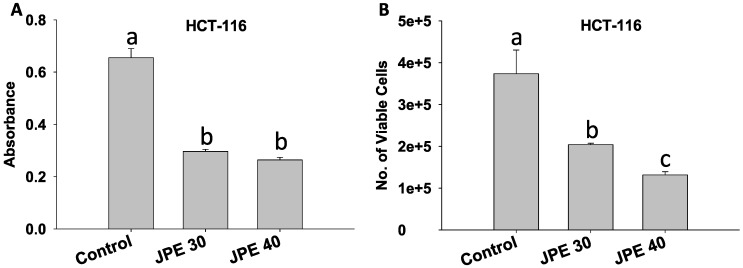
Java plum fruit extracts (JPE) suppressed proliferation in HCT-116 cells. HCT-116 cells were treated with JPE (30 or 40 µg/mL) for 24 hours, MTT assay (**A**) and viable cell count (**B**) were performed as described in methods. Values are in means ± SE. Means that differ by a common letter (a, b, c) differ at *p* < 0.05.

Beneath the complexity of every cancer lies critical events including deregulated cell proliferation, and suppressed apoptosis that provides a platform necessary to support further neoplastic progression. Cell proliferation is essentially an increase in the number of cells as a result of cell growth and cell division [29]. In our study, we evaluated the anti-proliferative effects of JPE using the MTT assay. There was a dose dependent suppression of cell proliferation in HCT-116 cells by JPE. At 30 µg/mL and 40 µg/mL (Figure 2A), there was suppression of proliferation (*p* < 0.05) by over 60% compared to control. Proliferation was also assessed by cell counting using an automated cell counter (Nexcelom) by treating the cells with JPE at 30 µg/mL and 40 µg/mL to confirm our observations with the MTT assay. Both concentrations resulted in more than 50% reduction in viable cell number (*p* < 0.05, Figure 2B). The suppression of proliferation by JPE in HCT-116 can be attributed to the presence of the identified compounds––anthocyanins, flavonols and stilbenoids, which have been previously shown to suppress proliferation in colon cancer cells individually and in combination, in *in vitro*, *in vivo*, and in human studies [12,30,31]. Previous studies with anthocyanin rich chokeberry extracts have shown that suppression of proliferation in HT-29 colon cancer cells occurs via cell cycle arrest [32]. Thus, further mechanistic studies are required to study molecular mechanism of anti-proliferative action of JPE against colon cancer cells, including its effect on proliferative pathways and the cell cycle.

### 2.3. JPE Induced Apoptosis in HCT-116 Cells and Colon CSCs

**Figure 3:**
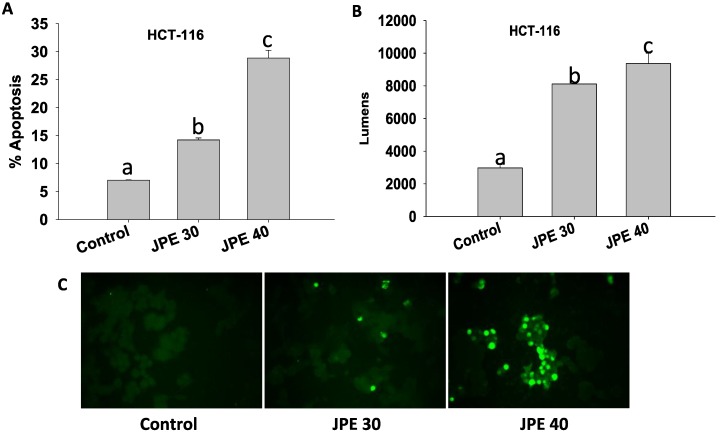
Java plum fruit extracts (JPE) induced apoptosis in HCT-116 cells; (**A**) Percent apoptosis in HCT-116 cells (n = 400) as measured by TUNEL assay. (**B**) Apoptosis was also assayed using caspase 3/7 glo assay. Values are in means ± SE. Means that differ by a common letter (a, b, c) differ at p < 0.05. (**C**) Cells fluorescing bright green due to fragmented DNA, indicator of apoptosis using TUNEL assay. Pictures were taken on a fluorescence microscope at 20× magnification (12 fields per treatment and at least 500 cells were counted). Representative pictures are shown for Control, JPE at 30 µg/mL and JPE at 40 µg/mL.

A hallmark of cancer is the ability of the cancer cells to evade apoptosis. Apoptosis can be seen as an important barrier to developing cancer; thus avoiding apoptosis is integral to tumor development and resistance to therapy [29]. In our study, we evaluated whether JPE extract can induce apoptosis in both HCT-116 colon cancer cells and colon CSCs. Induction of apoptosis was assayed by TUNEL assay, where fragmented DNA, characteristic of apoptotic cells, is used to identify apoptotic cells. Higher numbers of fluorescing cells indicate higher level of apoptosis (Figure 3C). JPE at 30 µg/mL and 40 µg/mL induced apoptosis (*p* < 0.05) in HCT-116 cells compared to control (Figure 3A,C). Further, apoptosis was also confirmed using Caspase 3/7 Glo assay. The assay measures the activity of caspases 3 and 7, which are responsible for fragmentation of DNA. JPE at 30 µg/mL and 40 µg/mL elevated (*p* < 0.05) caspase 3 and 7 dependent apoptosis in HCT-116 cells (Figure 3B) compared to control. Data from TUNEL and Caspase 3/7 Glo assay confirms that JPE induces apoptosis in colon cancer cell line HCT-116. The pro-apoptotic effect of JPE can be attributed to mitochondrial-mediated apoptosis, as the release of mitochondrial protein cytochrome c results in the step-wise activation of caspases ultimately leading to DNA fragmentation. Indeed, anthocyanin rich extracts of blueberries have been shown to activate caspase-3 in colon cancer cell line HT-29 [33].

**Figure 4:**
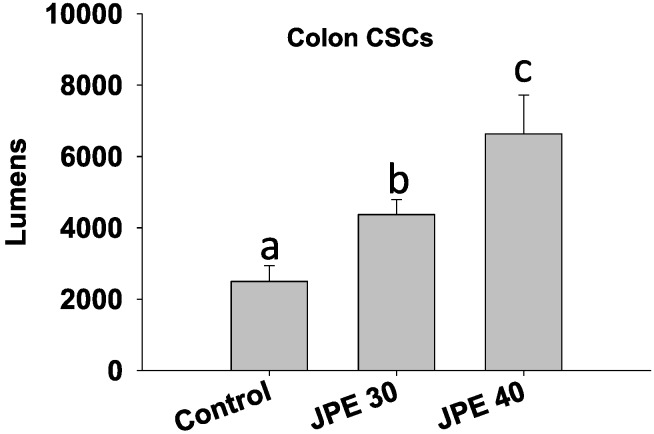
Java plum fruit extracts (JPE) induced apoptosis in colon cancer stem cells (colon CSCs). Cells were treated with JPE (30 or 40 µg/mL) for 24 hours and caspase 3/7 glo assay was performed. Values are in means ± SE. Means that differ by a common letter (a, b, c) differ at *p* < 0.05.

As colon CSCs are typically resistant to standard care therapies, we evaluated if JPE can induce apoptosis in colon CSCs using the Caspase 3/7 Glo assay. JPE at 30 µg/mL and 40 µg/mL induced apoptosis even in colon CSCs (*p* < 0.05) by more than 75% and 165% respectively compared to control (Figure 4). For the first time, we show that anthocyanin rich JPE extract induces apoptosis in human colon cancer cells HCT-116 and colon CSCs in a dose dependent manner. Our current results show that anthocyanin rich foods can be used to target CSCs via elevating apoptosis. In addition, recently curcumin—a major polyphenolic compound found in the Indian spice turmeric, was shown to synergistically act with chemotherapeutic drug—FOLFOX in elimination of colon CSCs [34]. Thus, further studies are required to evaluate anti-cancer properties of JPE alone or in combination with chemotherapeutic drugs. The combination approach helps in lowering the dosage, thus minimizing/eliminating side effects.

### 2.4. JPE Suppressed Colony Formation in Colon CSCs

**Figure 5:**
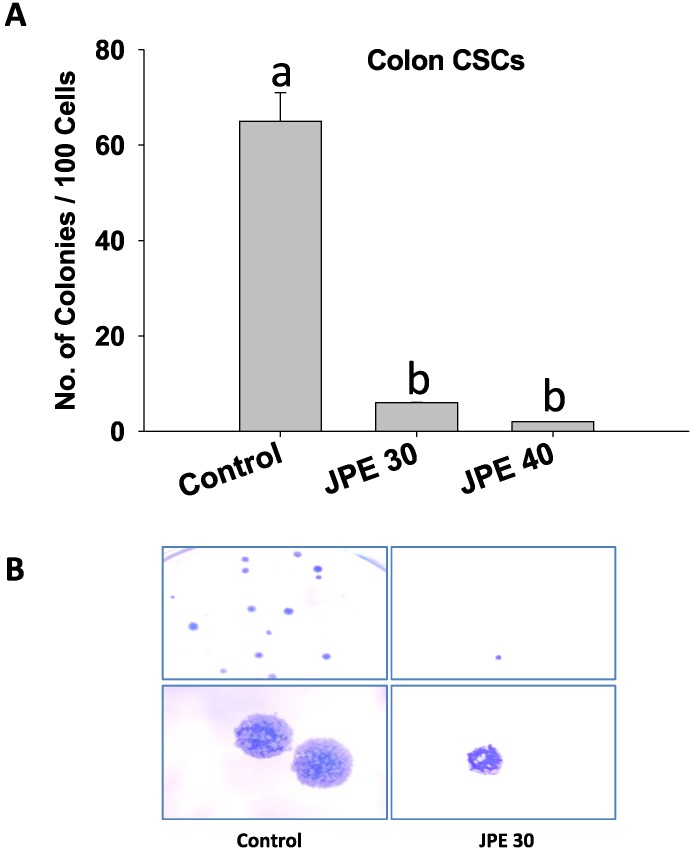
Effect of Java plum fruit extracts (JPE) on the stemness of colon CSCs. (**A**) Cells were treated with JPE (30 or 40 µg/mL) for 24 hours and colony formation assay was performed as described in methods. (**B**) Representative images taken from the colony forming assay for Control and JPE 30 are presented. Results were expressed as mean ± SE for three experiments at each time point. Means that differ by a common letter (a, b) differ at *p* < 0.05.

Colon CSCs possess the ability to initiate and drive the growth of tumors due to their self-renewal capability [35]. To assess the ability of JPE to target this capability, we used colony formation assay (Figure 5). Single cell suspensions of colon CSCs treated with JPE were grown in culture plates with complete growth media and the number of colonies was measured as described in the methods. Colon CSCs when treated with JPE at 30 µg/mL and 40 µg/mL respectively resulted in a dose-dependent suppression in colony formation (Figure 5A). Figure 5B also shows representative images collected from the colony forming assay and demonstrates the decreased colony number associated with JPE treatment compared to control. This shows that JPE affects colon CSCs self-renewal ability and thus demonstrates anti-cancer activities beyond suppressing proliferation and inducing apoptosis.

## 3. Materials and Methods

### 3.1. Extraction and Purification of Anthocyanins from Java Plum

The anthocyanin-rich fruit skin and pulp was carefully removed from the whole fruit and extracted with acidified methanol (0.1% HCl). The extract was concentrated under vacuum (40 °C) in a rotary evaporator for complete removal of the solvent. The concentrated extract was then dissolved in acidified water and partitioned with ethyl acetate to remove phenolics, flavonoids and/or carotenoid constituents. The aqueous extract was again concentrated under vacuum (40 ± 1 °C) to obtain anthocyanin concentrate. For further purification, anthocyanin-rich concentrate was adsorbed onto activated XAD-16 Amberlite resin column and eluted with 3 bed volumes of acidified water (0.1% HCl) to remove sugars, acids and/or other undesired water-soluble compounds. Anthocyanins adsorbed on the resin were subsequently eluted with acidified methanol. The methanolic extract was then concentrated in a rotavapor at 40 °C under vacuum. The resultant violet concentrate was dissolved in distilled deionized water containing 0.1% hydrochloric acid and lyophilized to get purified anthocyanin powder. The material was stored at −40 °C until further investigations.

### 3.2. Chemicals

Fetal bovine serum (FBS) was purchased from HyClone (Pittsburgh, PA, USA). All other chemicals and reagents were purchased from Sigma (St Louis, MO, USA).

### 3.3. High Performance Liquid Chromatography Mass Spectrometry (HPLC-MS) Analysis

JPE was dissolved in methanol to attain a concentration of 1 mg/mL. Anthocyanin profile of the powder concentrate was evaluated by a Waters Alliance HPLC (Pittsburg, PA, USA) equipped with Waters e2695 quaternary pump and 2998 photodiode array detector. Anthocyanin sample (20 μL injection volume; 1.0 mg/mL concentration) was analyzed on a Phenomenex (Torrance, CA, USA) RP-18 column (5 μM, 4.6 × 250 mm) using a gradient from solvent A (water, 0.1% trifluoroacetic acid (TFA)) to solvent B (water:ceric ammonium nitrate (CAN):TFA––53:46:1 v/v/v) at a flow rate 0.6 mL/min. Gradient: Initially at 20% B, then increased to 40% in 26 min, and thereafter to 80% in 4 min and held for additional 10 min. Anthocyanins were monitored at 520 nm.

The resulting column eluent was infused into a Micromass Q-Tof Micro MS fitted with an electrospray source (ESI) and analyzed for its constituents with the help of ESI-MS/MS spectrometer. Data was collected in positive ion mode, scanning from 50–1200 at a rate of 0.9 scans per second with 0.1 second interscan delay. Calibration was performed prior to sample analysis via infusion of sodium formate solution, with mass accuracy within 5 ppm. The capillary voltage was held at 2200 V, the source temp at 130 °C, and the desolvation temperature at 300 °C at a nitrogen desolvation gas flow rate of 400 L/hour. The quadrupole was held at collision energy of 7 V. Peak identities were obtained by matching their molecular mass [M]+ and MS/MS fragmentation ions as shown in Table 1 and Figure 1 and by comparison to published data [26]. ESI-MS has been successfully employed earlier for the characterization of bioactive azadirachtins in neem [22] and anthraquinones in Rheum emodi [23].

### 3.4. Cell Lines

Colon cancer cell lines HCT-116 were a generous gift from Bert Vogelstein (School of Medicine, Johns Hopkins University, Baltimore, MD, USA). Cells were maintained at 37 °C in a humidified atmosphere with 5% CO_2_ and grown in McCoy’s F-12 supplemented with 10% FBS, 2.2 g/L sodium bicarbonate, 0.2 g/L bovine serum albumin and 10 mL/L streptomycin-penicillin mix as described earlier [36].

Colon cancer stem cells (colon CSCs), positive for cancer stem cell markers CD 133, CD 44, and ALDH1b1, were obtained from Celprogen (San Pedro, CA, USA). To maintain the cells in their undifferentiated state, colon CSCs maintenance media and specially coated cell culture flasks obtained from Celprogen were used. Cells were maintained in incubation at 37 °C and 5% CO_2_ as described earlier [37]. Cell cultures at approximately 80% confluence were used for all *in vitro* experimental procedures.

### 3.5. Cell Viability

#### 3.5.1. MTT Assay

The cellular viability was evaluated using an assay based on the cleavage of the yellow dye MTT (3-(4, 5-dimethylthiazol-2-yl) 2, 5-diphenyl tetrazolium bromide) to purple formazan crystals by dehydrogenase activity in mitochondria. Briefly, 20,000 HCT-116 cells were seeded in a 96-well plate and after 24 hours, cells were treated with JPE at 30 and 40 µg/mL. After 24 hours, cells were rinsed with media and then they received MTT diluted in media for 4 hours as per the manufacturer’s protocol (Roche Diagnostics, Indianapolis, IN, USA). SDS/NaOH was used to dissolve the purple formazan crystals, and the optical density of the solution was measured at 570 and 690 nm. The experiment was performed in triplicate, and the data were expressed as the mean ± S.E.

#### 3.5.2. Cell Counting

Briefly, 100,000 HCT-116 cells were plated in a 12-well plate for 24 hours. They were treated with JPE at 30 µg/mL and 40 µg/mL. After 24 hours, 20 μL of the suspension were put in specialized slides obtained from Nexcelom Bioscience (Lawrence, MA, USA) and then inserted in Nexcelom automated cell counter. The experiment was performed in triplicate, and the data were expressed as the mean ± S.E.

### 3.6. Apoptosis

#### 3.6.1. Caspase Glo 3/7 Assay

Briefly, 100,000 cells (HCT-116 and colon CSCs) were seeded in a 12-well plate and incubated for 24 hours. They were treated with JPE at 30 and 40 µg/mL, after 24 hours, cells were trypsinized and approximately 20,000 cells from each treatment were incubated with 100 µL of Caspase Glo 3/7 reagent (Promega, Madison, WI, USA) for 30 min in a 96 well plate. The luminescence of each sample was measured using a BioTek micro plate reader (Winooski, VT, USA). The experiment was performed in triplicate, and data are expressed as means ± S.E.

#### 3.6.2. TUNEL Assay

Apoptosis was also assessed by terminal deoxynucleotidyl transferase-mediated dUTP nick end labeling (TUNEL) assay using an *in Situ* cell death detection kit from Roche Diagnostics. Experiments were carried out in accordance with the manufacturer's recommended procedures. Briefly, JPE treated HCT-116 cells grown on glass coverslips were fixed with 4% paraformaldehyde in PBS and were permeabilized with 0.1% Triton X-100 in 0.1% sodium citrate in PBS. They were then stained with the TUNEL reaction mixture and finally examined using a fluorescence microscope. At least 400 cells per treatment were counted and the results are expressed as percentage apoptosis (% ratio of apoptotic cells/total cells). The experiment was performed in duplicate and data are expressed as means ± S.E.

### 3.7. Colony Formation Assay

Ability of JPE to alter the stemness of colon CSCs was evaluated through colony formation assay [37] by counting the number of colonies that can form after treatment. Briefly, 150,000 colon CSCs were seeded per well in a 6-well plate and incubated for 24 hours in complete growth media. After 24 hours, growth media was removed and cells were treated with JPE at 30 µg/mL and 40 µg/mL for 24 hours. Cells were collected by trypsinization. One hundred treated cells were seeded into each well of a new 6-well plate and incubated for 10 days in complete growth media. At the end of 10 days, media was removed and cells were fixed using a fixing solution (3.7% paraformaldehyde in 70% ethanol) for 10 min. The cells were stained with 0.05% Coomassie blue for 20 min and then rinsed with PBS. Stained colonies were counted under a dissecting microscope as described earlier [38].

### 3.8. Statistical Analysis

Data were analyzed by one-way ANOVA using Tukey least square difference (LSD) with IBM SPSS software v22.0 (Armonk, NY, USA).

## 4. Conclusions

Bioactive compounds such as anthocyanins have shown potent anti-cancer effects in a variety of models [39,40]. Studies have also shown that anthocyanins selectively inhibit the growth of cancer cells with relatively little or no effect on the growth of normal cells [41]. Conventional cancer treatment options normally fail to specifically target CSCs leading to relapse in majority of the cases. In our study, anthocyanin-rich JPE clearly demonstrated anti-cancer properties not only against the early stage HCT-116 human colon cancer cells but also induced apoptosis and inhibited self-renewal ability in colon CSCs. The bioavailability of anthocyanins is low and hence reach the intestine at high concentrations and it has been suggested that concentrations in μg/mL dose range in the colon are feasible [42]. The number of *in vivo* studies demonstrating underlying mechanistic links to the anti-cancer/chemopreventive properties of phytochemicals derived from dietary foods is relatively low and thus, there is a need for higher number of studies on how dietary bioactive compounds can holistically help in preventing colon cancer. The promising results of our pilot study on JPE’s anti-cancer effects warrant future studies to evaluate the effect of JPE on CSCs *in vivo* utilizing animal models of colon cancer.

## Figures and Tables

**Table 1:** Anthocyanins present in JPE and their m/z vales from MS. Numbers correspond to peaks identified in chromatograph from Figure 1.

Peak	Anthocyanin	RT	[M]+	ESI-PI(m/z)
1	Delphinidin-3,5-diglucoside	12.68	627	465 [M-162]+; 303 [M-162-162]+
2	Cyanidin-3,5-diglucoside	14.44	611	449 [M-162]+; 287 [M-162-162]+
3	Petunidin-3,5-diglucoside	15.33	641	479 [M-162]+; 317 [M-162-162]+
4	Delphinidin-3-glucoside	16.22	449	287 [M-162]+
5	Peonidin-3,5-diglucoside	17.17	625	463 [M-162]+; 301 [M-162-162]+
6	Malvidin-3,5-diglucoside	17.83	655	493[M-162]+; 331 [M-162-162]+
7	Cyanidin-3-glucoside	18.74	449	287 [M-162]+
8	Petunidin-3-glucoside	19.26	479	317 [M-162]+
9	Unknown	20.28	-	-
10	Malvidin-3-glucoside	22.14	493	331 [M-162]+
11	Unknown	24.02	-	-
